# Solvodynamic Printing As A High Resolution Printing Method

**DOI:** 10.1038/s41598-019-47105-8

**Published:** 2019-07-24

**Authors:** W. C. Liu, A. A. R. Watt

**Affiliations:** Department of Materials, University of Oxford, 16 Parks Road, Oxford, OX1 3PH United Kingdom

**Keywords:** Surface patterning, Fluid dynamics

## Abstract

Printing techniques are becoming increasingly prevalent in modern manufacturing. However, its biggest drawback is the limit in printing resolution. In this paper, we present solvodynamic printing as a novel printing system which aims to improve print resolution by incorporating an additional immiscible carrier solvent into the ink delivery system. The resolution is improved due to the solvent-solvent interactions between the ink and the carrier solvent which alter the contact angle of the ink on the substrate and limit the printed feature size. We demonstrate the proof of concept of solvodynamic printing by printing silver nanoparticle inks on a polyethylene naphthalate substrate. Silver nanoparticle tracks with widths of 35.2 ± 7.0 μm were achieved using a 300 μm nozzle. This is equivalent to 11.7 ± 2.3% of the nozzle diameter. The result shows great potential in solvodynamic printing as not many modern printing techniques can achieve such nozzle to feature size ratios.

## Introduction

The patterning of functional materials has become an important aspect of manufacturing technology as it is essential in the fabrication of devices like microelectronic integrated circuits, microelectromechanical systems, optoelectronic devices and DNA microassays^1–5^. Traditionally, high resolution patterning is done via lithographic techniques which can achieve resolutions in the nanometer range^6^. However, such techniques are usually multi-step, require expensive equipment and low in throughput^7^. As a result, additive ink-based printing techniques like inkjet, flexographic and gravure printing have become increasingly used and sparked the development of the field of printed electronics. Such techniques have substantially lower costs and are compatible with large area high throughput processes^8–10^. In this article we use solvent-solvent interactions to create high resolution features for printed electronic applications using additive patterning techniques.

Among the family of additive printing techniques used in printed electronics, inkjet printing is one important method which has gained popularity due to its simplicity and low cost^11^. During operation, ink droplets are ejected from the printhead onto the desired location on the substrate and therefore do not require the use of masks or templates. It is also a purely additive and non-contact process, which minimizes material waste and substrate damage^12^. Although many electronic devices have been successfully fabricated by inkjet printing, its application is limited by its current resolution of around 20–30 µm^13^. Therefore, it cannot be used in many applications which require sub-micron feature sizes.

The resolution of inkjet printing is limited by the printhead nozzle diameter, the rheology of droplet formation and ink spreading on the substrate^13^. To further improve printing resolution, several different solutions have been developed. One such method is to pre-patterning the substrate structurally or chemically to alter the wetting behaviour of the ink droplets on different parts of the substrate^14,15^. However, the pre-patterning step generally requires another complex lithographic or mask-based high-resolution patterning technique prior to the printing step. This seems to undermine the overall advantage of using printing as a high throughput and simple patterning technique in the first place. Another way to improve the resolution is to reduce the dimensions of the ink droplets dispensed during printing. One such technique is electrodynamic inkjet printing which uses an additional electric field applied between the substrate and printhead to pull tiny droplets or jets from the printhead^16^. Electrodynamic inkjet printing has achieved resolutions as low as several microns. However, the addition of the electrical system adds to the complexity of the technique. Additional requirements on the ink’s electrical property must also be met as it affects the overall performance of the printing.

In this paper, we present a simple and novel solution to improve the printing resolution. The inspiration of this method is drawn from microfluidic microreactors, which synthesize materials within tiny droplets carried by an immiscible carrier solvent through a micron-scale channel^17^. We hypothesize that the incorporation of an immiscible carrier solvent into the ink delivery system can reduce the dimension of the ink ejected from printheads and alter the ink substrate interactions, leading to improvements in resolution. We name this technique “Solvodynamic printing” as the improvement in resolution arises from the molecular interactions between the ink and the immiscible carrier solvent. Here we demonstrate the proof-of-concept of solvodynamic printing by applying it to printing silver nanoparticle inks and focus on improving print resolution from a 300-micron nozzle.

## Solvodynamic Printing

### Printing schematics

In order to achieve good control over the fluid behaviour between two immiscible fluids, we employ the use of microfluidic chips in our printer setup. Microfluidic chips are devices with narrow channels used to manipulate the flow of small amounts of fluids^18^. Microfluidic chips are currently already used for many applications like fluid mixing, material synthesis and other biomedical applications^19–22^.

**Figure 1:**
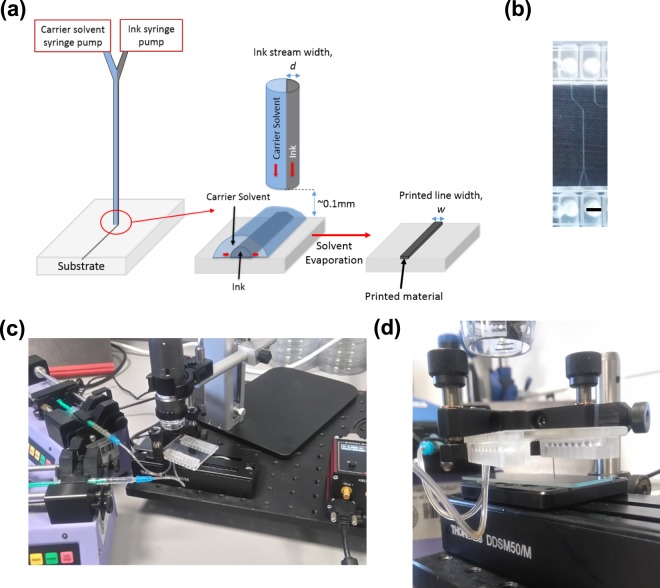
(**a**) Schematic of solvodynamic printing with relevant parameters. The carrier solvent is a solvent immiscible with the printing ink that is meant to improve printing resolution by reducing the size of the ink ejected from the printer and alter the wetting behaviour of the ink on the substrate. *d* denotes the width of the ink stream within the microfluidic channel and *w* denotes the final width of the dried printed line. (**b**) Design of microfluidic Y-junction chip. Scale bar represents 2 mm. Photographs of (**c**) the actual setup used in this study and (**d**) close-up of microfluidic chip and substrate configuration.

Figure 1a shows the schematic of our solvodynamic printing setup. Under operation, the carrier solvent and ink are pumped into a Y-junction microfluidic channel as shown in Fig. 1b through two inlets. Phase separation occurs when they meet due to their immiscible nature, causing the ink to flow alongside the carrier solvent through the channel. The fluids subsequently exit through the outlet nozzle of the chip and are deposited onto a substrate placed approximately 0.1 mm below the nozzle. After all the solvent has evaporated, the printed material will be left on the substrate. To control the printed pattern, the substrate is placed on a X-Y stage and moves relative to the stationary nozzle. Figure 1c shows the photographs of the exact setup used in this study.

### Controlling flow behaviour

The presence of the carrier solvent confines the ink to flow through a smaller part of the microfluidic channel and therefore creates a flow focusing effect on the ink stream^23^. This effectively reduces its stream width below the physical limits of the channels and nozzles. We hypothesize that this would have a beneficial effect on print resolution.

The flow focusing effect is easily controlled by the relative pump rates of the two fluids^24^. By increasing the carrier solvent pump rate (*q*_*c*_) relative to the ink pump rate (*q*_*i*_), the shear force exerted by the carrier solvent stream on the ink stream increases and deforms it to flow within a narrower region. We define the relative pump rate, $Q=\frac{{q}_{c}}{{q}_{i}}$. Figure S1 demonstrates the effect of altering *Q* on the resultant width of the ink stream. With increasing *Q*, the ink stream becomes narrower. A detailed explanation on the control of the ink flow within the microfluidic chip can be found in the Supplementary Information.

### Ink wetting behaviour

The incorporation of the carrier solvent also alters the ink wetting behaviour on the substrate after deposition. Given that the ink is denser than the carrier solvent, the carrier solvent will be able to surround the ink droplet on the substrate as shown in Fig. 1a. The presence of the carrier solvent around the ink alters the interactions between the ink and substrate^25^.

**Figure 2:**
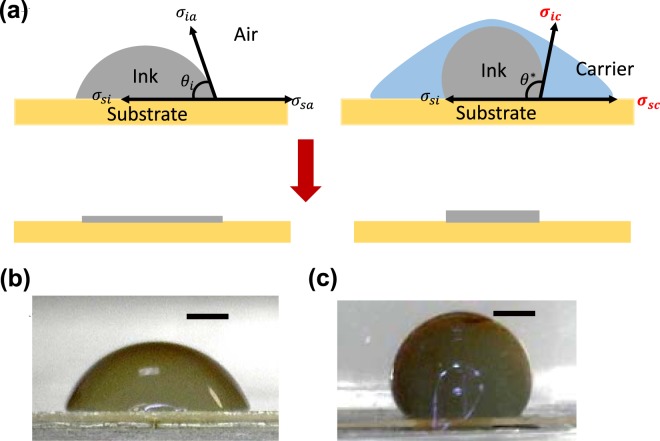
(**a**) Schematics of how changing wetting medium leads to improvements in resolution. The cross section geometry of the ink will be used to set up a model in the later section. *θ*_*i*_ and θ^*^ represent the equilibrium contact angle of the ink droplet in air and in carrier solvent respectively. *σ*_*ia*_*, σ*_*si*_*, σ*_*sa*_*, σ*_*ic*_ and *σ*_*sc*_ are the interfacial tensions between ink-air, substrate-ink, substrate-air, ink-carrier solvent and substrate-carrier solvent respectively. (**b**) Demonstrating the actual change in wetting behaviour of an aqueous silver nanoparticle ink droplet in air and (**c**) in hexane carrier solvent. Scale bar represent 0.1 mm.

The wetting behaviour of the ink, which is characterized by its contact angle, is important in printing as it is related to the area of contact between the ink and the substrate and therefore the dimensions of the resultant printed pattern^26^. The contact angle is determined by the interactions between three sets of interfacial energies as shown in Fig. 2a and is mathematically described by the Young’s equation^27^:

1 $$cos{\theta }_{i}=\frac{{\sigma }_{sa}-{\sigma }_{si}}{{\sigma }_{ia}}$$

where *σ*_*sa*_*, σ*_*si*_ and *σ*_*ia*_ are the substrate-air, substrate-ink and ink-air interfacial energies and *θ*_*i*_ is the resultant contact angle of the ink.

In solvodynamic printing, the wetting medium is changed from air to the carrier solvent. This changes two of the interfacial energies in the system and therefore alters the equilibrium contact angle of the ink. The Young’s equation can be modified to give:

2 $$cos{\theta }^{\ast }=\frac{{\sigma }_{sc}-{\sigma }_{si}}{{\sigma }_{ic}}$$

where θ* is the new equilibrium contact angle of the ink when completely surrounded by the carrier solvent. *σ*_*sc*_ and *σ*_*ic*_ are the interfacial tensions between the carrier solvent with the substrate and with the ink respectively.

Therefore, with the appropriate choice of carrier solvent, the contact angle can be increased which reduces the degree of spreading leading to higher printing resolution. Intuitively, this means that by choosing an appropriate carrier solvent which has more favourable carrier-substrate interactions than ink-substrate interactions, the three-phase contact line will shift to reduce the ink-substrate interface area, leading to higher print resolution. An example of this is shown in Fig. 2b,c. Figure 2b shows the profile of a droplet of aqueous ink on a substrate in air. When the medium is changed to a hexane carrier solvent, the contact angle increased drastically as shown in Fig. 2c.

### Mathematical model

Based on the concepts introduced in the previous section, we set up a basic mathematical model to relate the achievable resolution of solvodynamic printing to the printing parameters. We first make a basic assumption that the printed ink lines are infinitely long and have uniform truncated circular cross sections as in Fig. 2a. By considering the geometry of a circular segment, we can relate the width of the printed line to the contact angle, *θ* and the cross-sectional area of the segment, denoted by *A* as follows:

3 $$w=2sin\theta \sqrt{\frac{A}{\theta -sin\theta cos\theta }}$$

The cross-sectional area, *A*, can then be expressed in terms of ink loading per unit length along the printed line by considering the conservation of volume of ink:

4 $${\rm{A}}=\frac{{{\rm{q}}}_{{\rm{i}}}}{{{\rm{v}}}_{{\rm{s}}}}$$

where *v*_*s*_ is the substrate’s velocity beneath the nozzle. Combining (3) and (4), we get a combined expression (5) which relates the printing resolution to the printing parameters. An additional fitting parameter *k* is added to take into account any systematic mismatch between the modelled results and experimental results.

5 $$w=2ksin\theta \sqrt{\frac{{q}_{i}}{{v}_{s}(\theta -sin\theta cos\theta )}}$$

This preliminary set up will be combined with experimental results in the subsequent sections to obtain a final semi-empirical model.

## Results and Discussion

In this section, we set out to understand how different printing parameters affect the resultant resolution of solvodynamic printing. The three main parameters of interest are the ink stream width within the microfluidic channel, ink surface tension and substrate surface energy.

### Ink stream width

**Figure 3:**
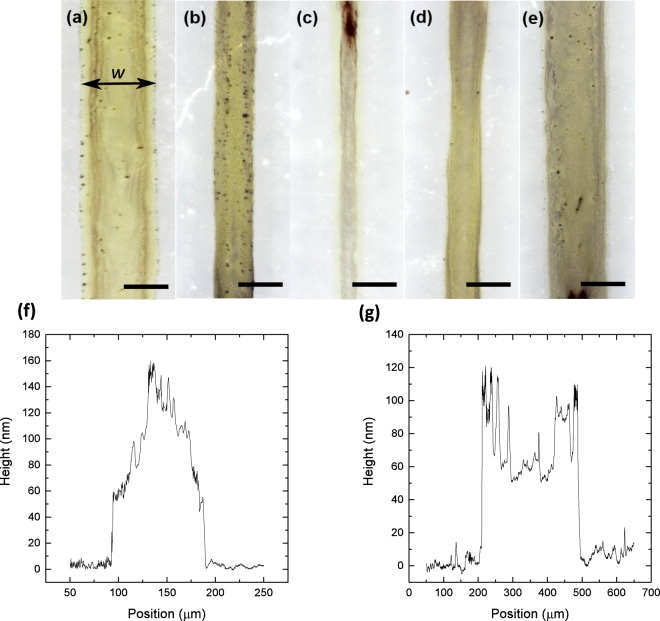
Micrographs of solvodynamic printed lines with widths denoted by *w*. They were printed while varying the ink stream width, *d* within the microfluidic channel. Lines were printed with *d* = (**a**) 200 µm, (**b**) 157 µm, (**c**) 125 µm, (**d**) 41 µm, (**e**) 34 µm. *d* was varied by adjusting the relative pump rate of carrier solvent to ink, *Q*. The ink pump rate was fixed at 50 µl/hr, ink consisting of silver nanoparticles with concentration of 6 mg/ml dispersed in water with 15 wt% of ethylene glycol. The substrate used is polyethylene naphthalate which was ozone-treated for 3 min. Substrate velocity was set at 20 mms^−1^. Scale bars represent 200 µm. (**f**,**g**) are cross sectional profile of a solvodynamically printed line and a line printed without carrier solvent respectively.

Our main hypothesis regarding solvodynamic printing is the improvement in resolution by focusing the flow of ink into a narrower stream. Therefore, we first study how the width of the ink stream within the microfluidic channel, denoted by *d*, affects the width of the final printed line, denoted by *w*. By controlling the relative pump rate *Q*, silver nanoparticle ink streams with widths, *d* ranging from 34 to 200 µm were used to print silver lines on the substrate. Figure 3a–e shows the micrographs of a set of printed lines using different *d*. Figure 3a shows the control line which was printed without the use of carrier solvent (*Q* = 0 and *d* = 200 µm). Without the use of carrier solvent, a printed line width of approximately 300 µm was achieved. As *Q* was increased and *d* decreased, we observed that the printed lines became thinner. This shows the proof-of-concept of solvodynamic printing as the use of carrier solvent improved the printing resolution. The value of *w* decreased to a minimum before increasing again at lower values of *d*.

It is noted that since hexane is much more volatile than water, we would expect the carrier solvent to evaporate before the ink fully dries. In addition, the ink should only start drying after the surrounding carrier solvent has evaporated. If this was the case, the carrier solvent should have had no effect on improving the printing resolution. Therefore, it is likely that the contact line has been pinned by the silver nanoparticles upon the initial deposition. This is a common phenomenon in liquids with solutes or dispersed particles, which accumulate at the three-phase contact line due to inherent surface roughness or heterogeneity of the substrate^28^. As a result, the width of the printed lines remains fixed even after the carrier solvent has evaporated. The uneven cross section profile of a printed line as shown in Fig. 3f supports the idea of contact line pinning. The profile shows that the middle of the line has accumulated more nanoparticles compared to the sides. We believe that this could be due to the drying characteristics of the printed lines. During the initial deposition, most of the hexane was accumulated near the bottom of the deposited ink due to gravity, leaving a thin layer of hexane around the top of the ink. Therefore, it is likely that the hexane at the top dried the quickest to expose the ink, causing the ink drying to begin from the middle of the printed lines towards the edges^29^. Conversely, the profile of the control line in Fig. 3g shows more nanoparticles on the sides compared to the middle, which is a typical profile of a contact line pinned feature drying under normal conditions.

**Figure 4:**
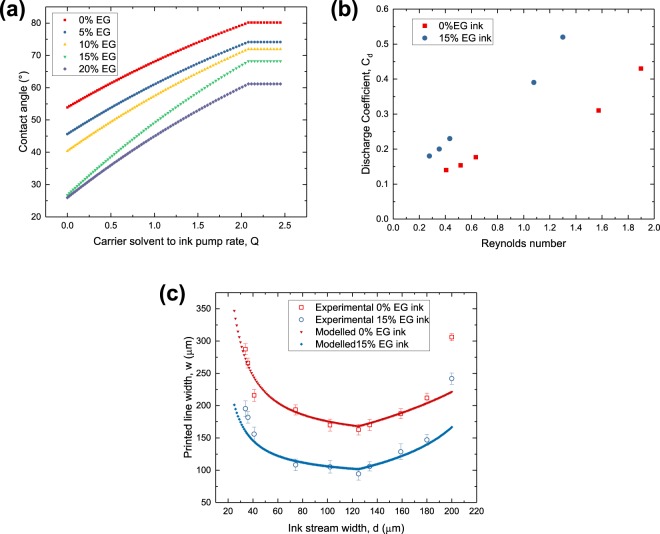
(**a**) Approximated variation of ink contact angle with varying ratios of carrier solvent. Contact angle reaches maximum value when volume of carrier solvent is approximately double of volume of ink. (**b**) Experimental results of discharge coefficient against Reynolds number of the ink, which is varied by the relative pump rate of carrier solvent to ink. (**c**) Experimental and modelled variation in printed line width (*w*) with the change in ink stream width (*d*).

From the model Eq. (5), we can see that the contact angle is one of the main parameters in determining *w*. Therefore, we start by understanding the relation between the ink stream width, *d* and the contact angle of the ink, *θ*. We recall that the ink stream width is controlled by the ratio of carrier solvent to ink pump rate. Therefore, variations in *d* is related to the ratio of carrier solvent to ink deposited. Theoretically, we know that the contact angle increases from an initial *θ*_*i*_ in air to a new equilibrium, θ* under the carrier solvent. Additional experiments were done to elucidate how this change occurs. We monitored the contact angle of ink droplets as we gradually added hexane onto them. It was observed that the contact angle did not change abruptly from *θ*_*i*_ to θ* once the carrier solvent was added. Instead, there was an initial range whereby the contact angle increased gradually with the amount of hexane added until the maximum of θ*, beyond which no further changes occurred with further addition of carrier solvent. Unfortunately, the exact way which this increase occurred was difficult to observe. Therefore, we constructed linear approximations from the *θ*_*i*_ and θ* for each combination of inks and substrates to model the behavior between *d* and *θ*. This is depicted in Fig. 4a. The approximations were then used in Eq. (5) to model the variations in w caused by *d*. The initial increase in *θ* corresponds to the decrease in *w* observed when *d* decreases from 200 μm to 125 μm as the term $\frac{sin\theta }{\sqrt{\theta -sin\theta cos\theta }}$ is decreasing in *θ*.

To model the increase in *w* at lower values of *d*, we considered several other possible factors. We first noticed that a lower *d* corresponded to a higher ink velocity since the ink stream had a smaller cross-sectional area. Therefore, we considered the effects of additional spreading or splashing during the ink deposition due to a higher ink velocity from the nozzle by using a model by Ukiwe *et al*.^30^. However, the calculated variations in *w* were too small to explain the large variation observed from the experiments. In addition, the low *Re* and *We* of the system meant that the ink velocities were not high enough to overcome the viscosity and surface tension forces to generate this effect^31^. Next, we also considered the possibility of the ink droplet forming a more elliptical or paddle-shaped cross section under additional pressure from the additional hexane^32,33^. This was also dismissed upon further investigation as numerical simulations did not produce sufficient variation in *w*.

Finally, we realized that the amount of ink dispensed was not constant which altered the ink pump rate *q*_*i*_, another parameter in the model equation. This is related to the discharge coefficient, *C*_*d*_ of the printer. The discharge coefficient is the ratio of the actual fluid discharge to the theoretical discharge of a fluidic system^34^. This mainly applies to systems which involves continuous stream or jets of fluids. The presence of the nozzle and constrictions from the connectors on the microfluidic chip reduces the flow rate of the fluids. Prior literature has established that the discharge coefficient has a positive relation with the Reynolds number of the fluid^34,35^. Since a lower *d* is related to a higher ink flow rate and Reynolds number, the discharge coefficient will be higher at lower values of *d*. To verify this effect, we performed separate experiments to measure the amount of ink dispensed by the printer over a prolonged period at a fixed ink pump rate with different ink stream widths. Indeed, we observed that more ink was being dispensed as the ink stream width was decreased as shown in Fig. 4b. Since *q*_*i*_ is no longer constant, we modify the model equation by including *C*_*d*_.

6 $$w=2ksin\theta \sqrt{\frac{{C}_{d}{q}_{i}}{{v}_{s}(\theta -sin\theta cos\theta )}}$$

The empirical data from these experiments were then included in the main solvodynamic printing model and are shown in Fig. 4c. We can clearly see that the overall interactions between variations in *θ* and *C*_*d*_ at different values of *d* matches well with the experimental results.

From the model, we can see that the improvement in resolution is largely caused by the increasing contact angle of the ink in the presence of the carrier solvent. A further observation was that *k* had to be varied when fitting the modelled data for inks with different compositions. This would be investigated in the next section.

### Ink surface tension

The second parameter of study is the ink surface tension. Inks with higher surface tension leads to larger contact angles and therefore less wetting and higher resolution can be achieved^25^. However, when the printed features are lines instead of droplets, the surface tension plays another role. Lines of ink deposited onto the substrate during printing are inherently unstable due to higher total interfacial energy between the ink and its surroundings. They tend to break up into more energetically stable droplets^36,37^. Therefore, there is an optimal surface tension for inks to achieve high resolution and good stability.

**Figure 5:**
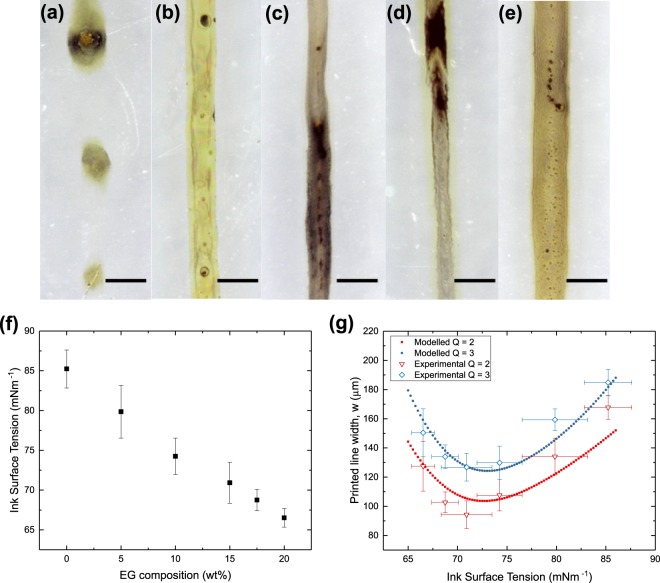
Micrographs of solvodynamic printed lines using inks with different surface tensions by varying the amount of ethylene glycol mixed into the ink: (**a**) 0 wt%, (**b**) 5 wt%, (**c**) 10 wt%, (**d**) 15 wt%, (**e**) 20 wt%. The ink and carrier solvent pump rate was fixed at 50 µl/hr and 100 µl/hr respectively, inks consisted of silver nanoparticles with concentration of 6 mg/ml. The substrate used was polyethylene naphthalate which was ozone-treated for 3 min. Substrate velocity was set at 20 mms^−1^. Scale bars represent 200 µm. (**f**) Variation in polyethylene naphthtalate substrate surface energy with the change in duration under ozone plasma treatment. (**g**) Experimental and modelled variation in printed line width (*w*) with the change in ink surface tension.

In our study, the ink surface tension was controlled by mixing ethylene glycol (EG) as an ink co-solvent. EG was chosen as it is water soluble but immiscible with the hexane carrier solvent, and has a lower surface tension than water. Several inks with different EG-water composition were made and their calculated surface tensions are displayed in Fig. 5f. As expected, the ink surface tension decreased with increasing amount of EG.

The different inks were then used to print lines using *Q* = 2 and 3, which achieved high resolutions from the previous experiments. Figure 5a–e shows the micrograph of the printed lines and the numerical data is tabulated in Fig. 5g. We first observe that some of the lines printed with the purely aqueous ink were unstable and broke up into droplets as shown in Fig. 5a. This probably indicates that the surface tension of this ink was too high and formed unstable lines. As the surface tension decreased, more stable and longer lines could be formed. At the same time, the line widths decreased to a minimum before increasing again. This observation is quite interesting as lower surface tensions tend to cause increased wetting and *w* should increase. Therefore, it is likely that other parameters in the printing process were also contributing to changes in *w*.

**Figure 6:**
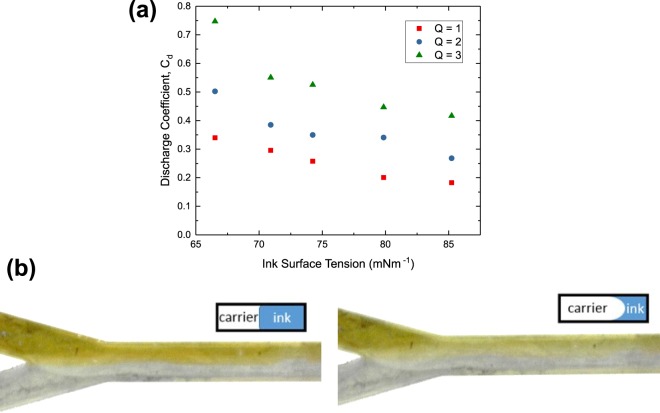
(**a**) Observed variation in discharge coefficient of the printer when using inks with different surface tensions. (**b**) Micrographs of ink flow in microfluidic channels using high surface tension ink with 0 wt% EG (left) and low surface tension ink with 20 wt% EG (right) at the same pump rate ratio. Inset diagrams are the schematics of the cross-sectional shape of ink stream for the respective micrographs.

From our experiments on the discharge coefficient of the printer, it was found that the discharge coefficient actually decreased with increasing ink surface tension shown in Fig. 6a. The relation between surface tension and discharge coefficient is not widely studied in current literature. Nevertheless, we postulate that this observation is related to the relation between Reynolds number and discharge coefficient. Numerical studies on multiphase flows from the literature show that the change in surface tension of one of the fluids, which in turn changes the interfacial tension between the fluids, can alter the shape of the interface between the two fluids^38^. An increase in interfacial tension tend to create a straighter interface to reduce the overall interfacial tension while a decrease in interfacial tension will create a more curved interface as shown in the insets of Fig. 6b. Therefore, when surface tension is increased, the ink-carrier interface changes from a curved interface to a straighter one. This increases the cross-section area of the ink stream and reduces the velocity of the ink flow. As a result, the Reynolds number of the ink stream decreases and causes the discharge coefficient to decrease. However, this is difficult to verify as the actual cross-sectional shape of the ink flow within the microfluidic chip cannot be easily characterized. However, images of ink flow in the microfluidic chips seem to support our explanation. Figure 6b shows micrographs of the microfluidic channels with 0 wt% EG and 20 wt% EG inks flowing through. A faint difference in the transparency of the ink stream was observed and this could be due to the different cross section shapes.

**Figure 7:**
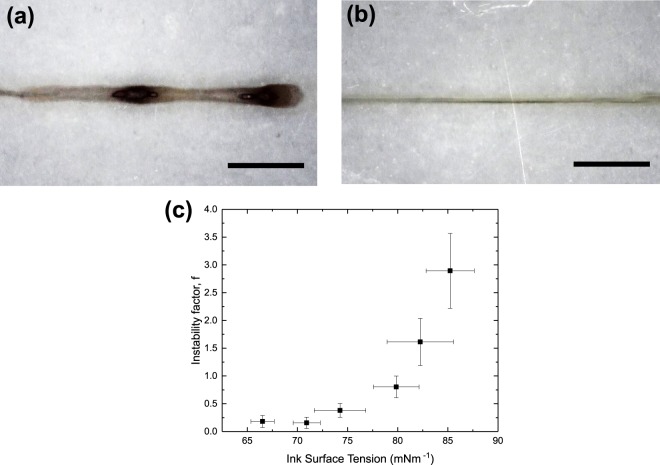
Low Magnification micrographs of printed lines using (**a**) 0 wt% EG ink and (**b**) 20 wt% EG ink. The scale bar represents 500 μm. (**c**) Variation of the instability factor, *f* against ink surface tension. *f* is defined as the ratio of the lengths between separate printed features to the lengths of each feature.

From the previous section, we observed that the fitting constant *k* changed with the different inks used as shown in Fig. S3. Upon further investigation, we believe that this is related to the formation of unstable lines as mentioned earlier. An example is shown in Fig. 7a. Interestingly, the unstable lines were largely associated with ink surface tensions rather than substrate surface energies. This led us to think that this was an effect associated with our printer rather than the lines breaking up after deposition on the substrate. A reasonable explanation would be related to the dispensing of the inks from the nozzle. It is more difficult for high surface tension inks to flow out of a nozzle as a stream. Therefore, it may have accumulated into a larger droplet before dropping onto the substrate. This increases the volume of ink per unit length and *w*. To include this effect into the model, we defined a new parameter which we call the instability factor as the average ratio of the lengths between each consecutive printed features to the lengths of each printed feature. The experimental variation between the instability factor, denoted as *f* and the ink surface tension is shown in Fig. 7b. Mathematically, the volume of the ink in each printed feature would then increase by a factor of (1 + *f*). This factor was then included in our model in place of the original fitting parameter *k* to obtain the finalised form in Eq. 7. This finalised model shows good match with the experimental results as shown in Fig. 5g.

7 $$w=2sin\theta \sqrt{\frac{(1+f){C}_{d}{q}_{i}}{{v}_{s}(\theta -sin\theta cos\theta )}}$$

### Substrate surface energy

The last parameter studied was the substrate surface energy. Generally, liquids are able to wet surfaces with higher surface energies better. Therefore, low surface energy substrates are used to achieve high resolution features. However, similar to the case for surface tension, substrates having too low surface energy will lead to instability in the printed lines. The effect of substrate surface energy on solvodynamic printing resolution should be quite predictable as it only affects the contact angle. Increasing the surface energy of the substrate will lead to increase in wetting of the ink and lead to poorer resolution.

**Figure 8:**
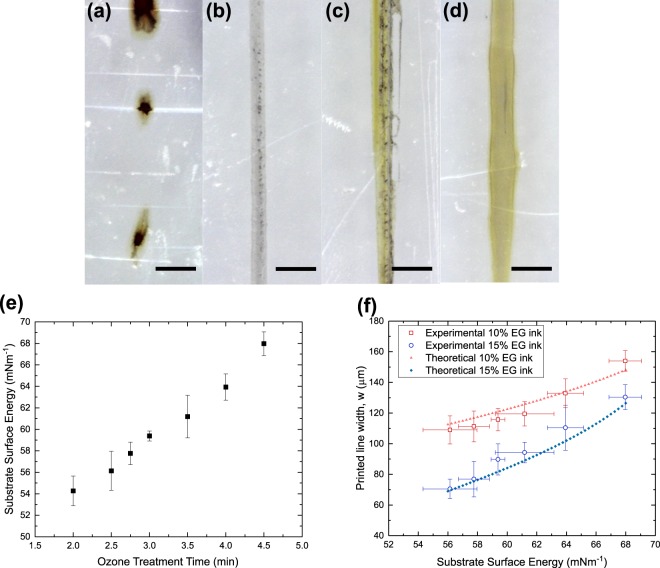
Micrographs of solvodynamic printed lines using polyethylene naphthalate substrates treated by ozone plasma for different times: (**a**) 2 min, (**b**) 2.5 min, (**c**) 3.5 min and (**d**) 4.5 min. The ink and carrier solvent pump rate was fixed at 50 µl/hr and 100 µl/hr respectively, ink consisted of silver nanoparticles with concentration of 6 mg/ml dispersed in water with 15 wt% of ethylene glycol. Substrate velocity was set at 20 mms^−1^. Scale bars represent 200 µm. (**e**) Variation in substrate surface energy with the change in ozone plasma treatment time. (**f**) Variation in printed line width (*w*) of 2 sets of printed lines with change in substrate surface energy.

In our study, the PEN substrate’s surface energy was controlled by subjecting it to an ozone plasma treatment for different durations. The plasma treatment was necessary as the inks could not wet the untreated substrates at all. Figure 8e shows the variation of the PEN surface energy with different plasma treatment durations. Figure 8a–d shows a set of micrographs of the silver nanoparticle lines printed on substrates with varying substrate energy using the 15 wt% EG ink and relative pump rate of *Q* = 2, which were found to be optimal in previous sections.

From Fig. 8a, we first observed that the sample with the lowest surface energy could not form stable silver lines due to the instability as discussed above. For the rest of the samples which could form stable lines as in Fig. 8b–d, we observed that the printed line width increased with substrate surface energy as predicted. Figure 8f shows the overall variation of line widths with changing substrate energy for 2 sets of printed lines. Once again, the modelled data showed good match with the experimental results.

**Figure 9:**
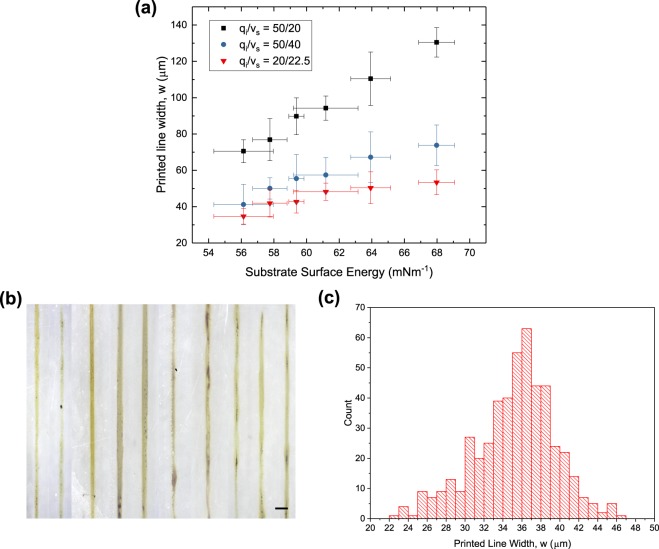
(**a**) Variation in printed line widths with substrate surface energy for 3 sets of lines printed with different ink pump rate (*q*_*i*_) to substrate velocity (*v*_*s*_) ratios. Values of *q*_*i*_ and *v*_*s*_ are measured in μl/hr and mms^−1^ respectively. The carrier solvent to ink pump rate was fixed at 2 and the ink used with 15 wt% EG and 6 mg/ml silver nanoparticles. (**b**) Optical images of 10 printed lines at the optimized parameters. Scale bar represents 100 μm. (**c**) The distribution of the line widths taken at points every 40 μm along the lengths of the 10 printed lines for 50 points per line.

To further minimize the line widths to achieve the lowest resolution, we proceeded to adjust the ink pump rate to substrate velocity ratio, $\frac{{q}_{i}}{{v}_{s}}$ as seen in the model. The ratio of these parameters affects the ink loading per unit length of the printed lines. The results are shown in Fig. 9a. We initially attempted to increase the substrate velocity up to 40 mms^−1^ while keeping the ink pump rate as 50 μl/hr. Thinner lines of approximately 40 μm were observed but the high substrate velocity seemed to be detrimental to the consistency of the system as the variance of the printed line widths increased dramatically. Therefore, the substrate velocity was lowered together with the ink pump rate to keep the ink loading low while avoiding high substrate velocities. We eventually settled on an ink pump rate of 20 μl/hr and substrate velocity of 22.5 mms^−1^. At these parameters, lines of approximately 35 μm with reasonable variance were printed. Any further decrease in the ink pump rate to substrate velocity ratio could not produce continuous lines probably due to the ink loading being insufficient.

To determine a reliable value of the attainable resolution of solvodynamic printing, we employed a statistical approach and examined ten line segments printed using the optimized parameters as shown in Fig. 9b. The widths were measured at points every 40 μm along the lines for 50 points per line. The distribution of the widths are then tabulated into a histogram as shown in Fig. 9c. The average width is 35.2 μm with a standard deviation of 4.2 μm. As a statistical estimation, 90% of the printed line widths will fall within the range of 35.2 ± 7.0 μm. This is equivalent to 11.7 ± 2.3% of the nozzle diameter.

## Conclusion

In this paper, we have shown the proof-of-concept of using solvent interactions between the ink and an additional immiscible solvent as a mean to improve printing resolution. The improvement in printing resolution was primarily due to the increase in the ink contact angle with the substrate. The relation between different printing parameters and the achievable resolution was studied and a model was developed to explain the observed results. Using this technique, lines of silver nanoparticles with widths of approximately 35.2 ± 7.0 μm were achieved using a 300 μm diameter nozzle, equivalent to 11.7 ± 2.3% of the nozzle diameter. This result is promising and shows that there is still much potential for this technique to improve. With further downsizing of the system, even higher resolution can possibly be achieved. In addition, this technique may also be compatible with other resolution-improving techniques to further boost the achievable resolution of additive printing technology.

## Materials and Methods

### Materials

Silver nitrate, ethylene glycol, sodium chloride and polyvinylpyrrolidone (PVP) with average molecular weight of 55000 are purchased from Sigma Aldrich.

### Methods

#### Formulation of silver nanoparticle ink

Silver nanoparticles used in this study were synthesized via the polyol process^39^. In a typical synthesis, 0.0955 g of silver nitrate was dissolved in 0.75 ml of ethylene glycol. In a separate glass vial, 1.0 g of PVP was dissolved in 10 ml of ethylene glycol and heated to 135 °C with magnetic stirring. 20 µL of a 0.15 M aqueous sodium chloride solution was then added to the PVP solution. Once the solution is mixed homogenously, the silver nitrate solution was added and left for 1 hour. After the reaction, the nanoparticles were washed with methanol and centrifuged at 6000 rpm. This is repeated 3 times before finally redispersing the nanoparticles into water to form an aqueous ink.

#### Solvodynamic printer set-up

Two syringe pumps (Harvard Apparatus Model ‘11’ Plus 70-2208) were used to inject the silver nanoparticle ink and carrier solvent into a microfluidic Y-junction chip (Cellix Ltd) which has a rectangular channel with width of 200 µm and depth of 50 µm with a hydrophobic wall coating. The fluids exited the chip through a 300 µm inner diameter outlet pin which also acted as a nozzle. The pin was positioned 100 µm above a polyethylene naphthalate (PEN) substrate which was placed on a computer-controlled translational stage (Thorlabs DDSM50). The substrates were treated with ozone-plasma prior to the printing using UV-Ozone cleaner (Ossila). After the printed silver nanoparticle lines were dried on a hotplate at 110 °C for 10 mins.

#### Characterization

The dimensions of the printed features and the ink stream width within the microfluidic channel were characterized by a digital microscope (DinoLite). The cross section profile of the printed lines were obtained using a Veeco DekTak 6M stylus profiler. The ink surface tension and substrate surface energy were determined by taking contact angle measurements and analyzing them using the Equation of States approach developed by Kwok and Neumann^40^.

## Supplementary information

Supplementary Information

## Data Availability

The primary dataset used to generate the figures in this manuscript can be accessed here: https://figshare.com/s/0a7377d70a90a302e952.
