# Supplementary material for: Solvodynamic Printing As A High Resolution Printing Method
Source: Sci Rep. 2019 Jul 24;9:10766. doi: 10.1038/s41598-019-47105-8 (PMC6656777; doi:10.1038/s41598-019-47105-8)
Supplement: Supplementary file 1 — Supplementary Information [file 41598_2019_47105_MOESM1_ESM.docx]

Title: Solvodynamic Printing As A High Resolution Printing Method

**Authors**: W.C. Liu^1^, A.A.R. Watt^1*^

Affiliations: ^1^Department of Materials, University of Oxford, 16 Parks Road, Oxford OX1 3PH, United Kingdom.

*Correspondence to: [Andrew.watt@materials.ox.ac.uk](mailto:Andrew.watt@materials.ox.ac.uk)

**Supplementary Information**

1. Supplementary Discussion

**Microfluidics flow characteristics**

The flow characteristic of the fluids within the microfluidic chip is an important part of solvodynamic printing as it determines the configuration of the ink-carrier mixture as it leaves the printer nozzle. The fluid behaviour can typically be characterised using different dimensionless numbers like the Weber (*We*) and Capillary (*Ca*) numbers as shown below^41^.

$We= \frac{\rho V^{2}L}{\sigma}$ (7)

$Ca= \frac{\mu V}{\sigma_{ic}}$ (8)

where *ρ* is the fluid density, *V* is the fluid velocity, *L* is the characteristic length of the flow, *µ* is the fluid dynamic viscosity and *σ_ic_* is the interfacial energy between the two fluids.

The flow of two immiscible fluids in the chip can occur in two main ways: droplet and parallel flow as shown in Figure S2^42^. The type of flow which occurs is determined by the balance of the interfacial tension between the ink and carrier solvent and the viscosities of the fluids. This is typically characterized by the Capillary number^43^. Low Capillary numbers indicate that the interfacial energy between the fluids dominates the viscous forces. This promotes the formation of ink droplets to reduce the overall interphase area and minimize interfacial energy of the system. On the other hand, high capillary numbers indicate that viscous forces dominates, allowing the ink stream remains intact through the channel and forming a parallel flow. Some studies also found relationships between flow regime and the Weber numbers of the two fluids as higher flow rates tend to stabilize parallel flow^44^. The exact conditions which causes a transition between the droplet and parallel flow regime is not well-understood. However, it is established that the transition is strongly dependent on the junction shape, fluid pump rate ratio, viscosity ratios aside from the Capillary and Weber numbers^45,46^.

Both flow regimes can be used to perform solvodynamic printing with the droplet formation regime behaving like a droplet-on-demand printing system while the parallel flow regime behaves more like a jetting mode^45^. However, we have chosen to focus on making use of the parallel flow regime as we believe that it would be more suitable for printing elongated features which are most common in printed electronics applications.

1. Supplementary Figures

**(c)**

**(b)**

**(a)**


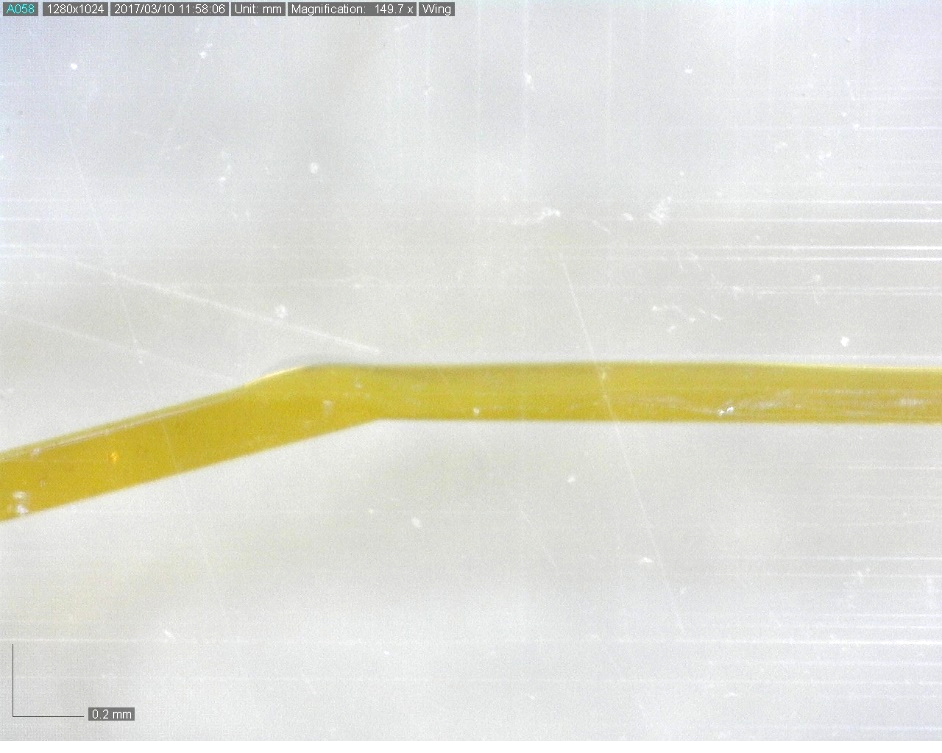

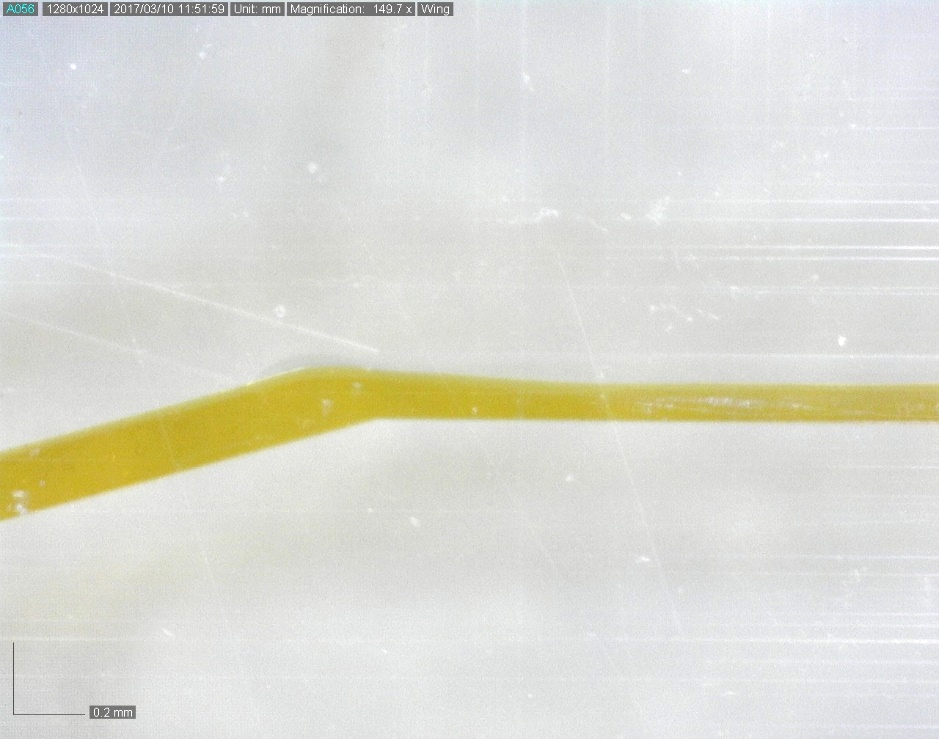

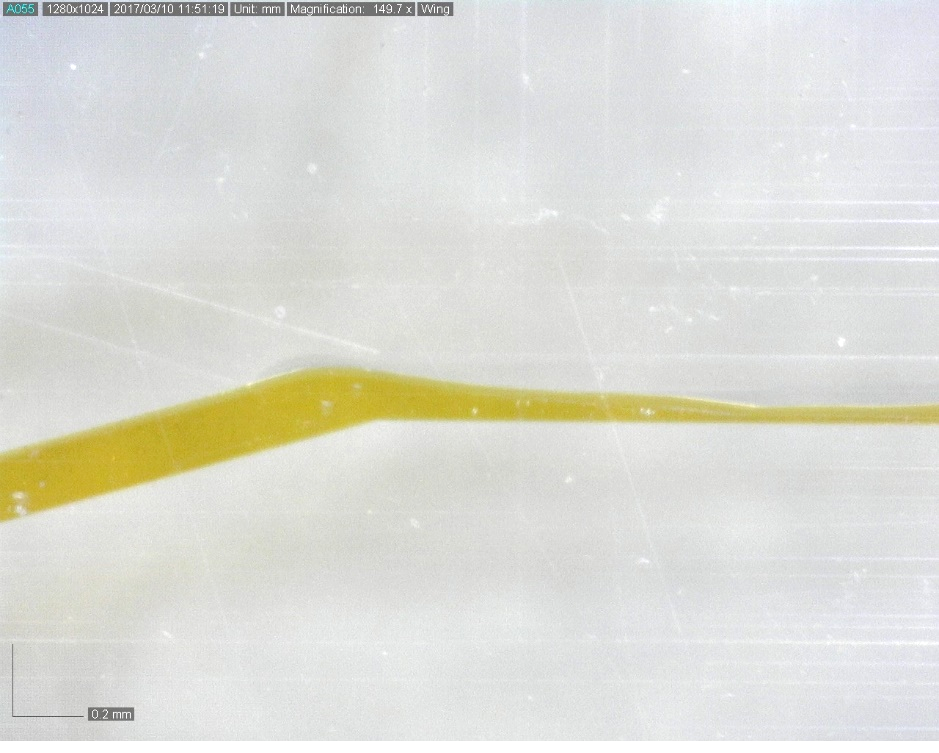

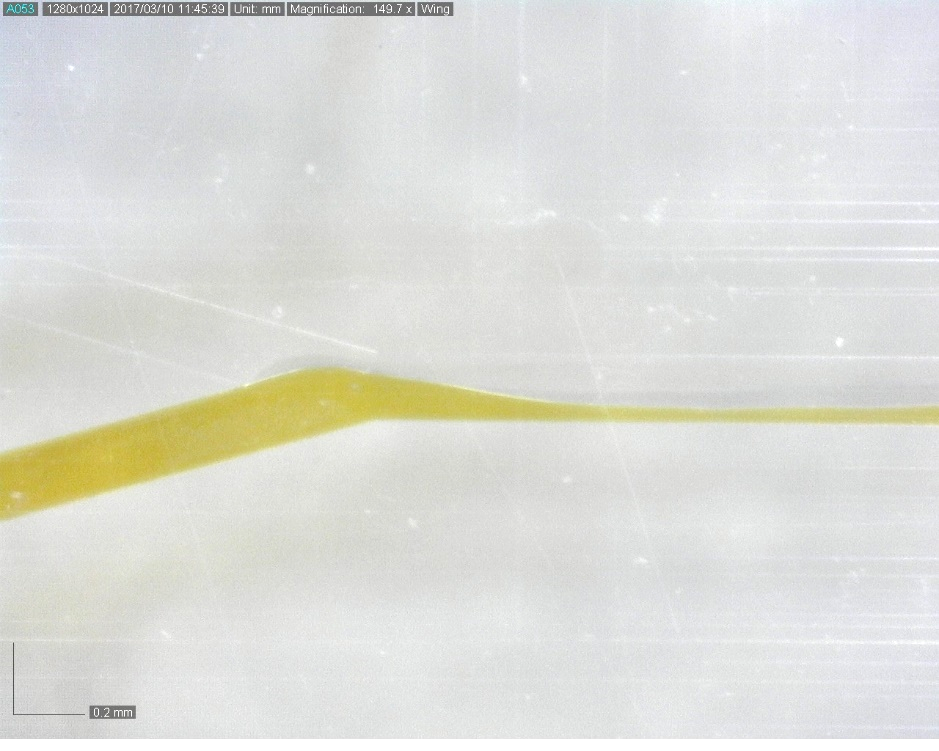


**(d)**

**Fig. S1**: Micrograph of fluid carrier solvent (clear) and ink (coloured) flow within the microfluidic chip at different relative pump rates, *Q*. (a) *Q* = 1 (b) *Q* = 3 (c) *Q* = 5 (d) *Q* = 8. The scale bar represents 200 μm.


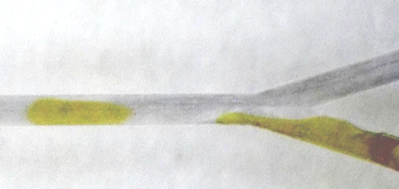

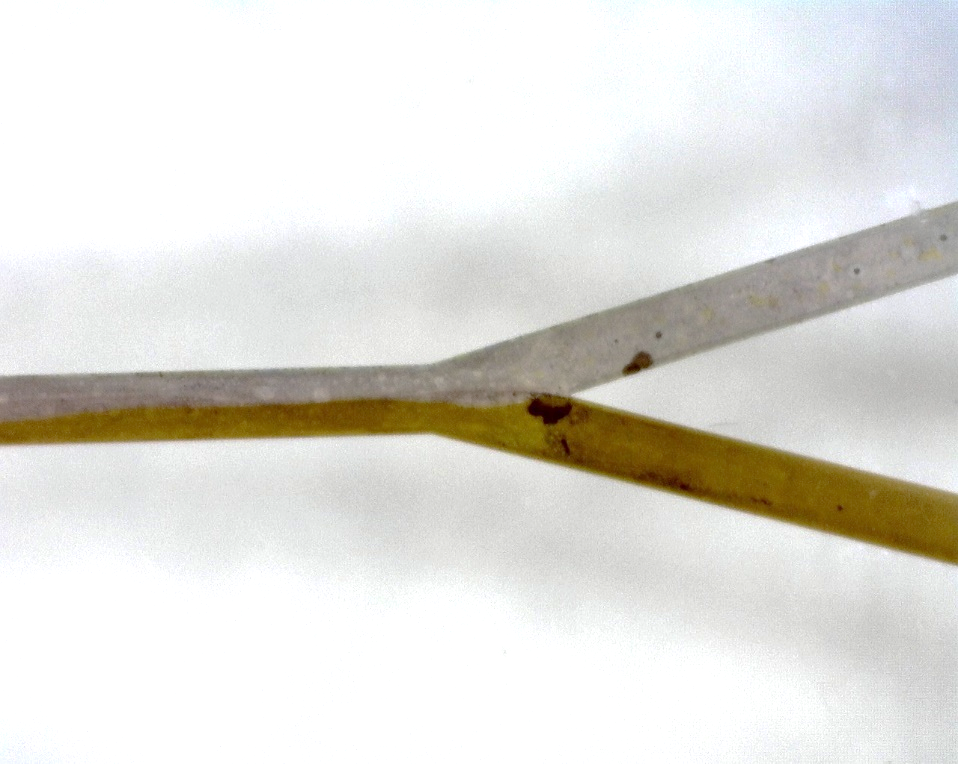


**(b)**

**(a)**

**Fig. S2**: Different flow regimes for two immiscible fluids: (a) Droplet formation and (b) Parallel flow.

Fig. S3: Variation of the fitting parameter k, against ink surface tension.

**Supplementary References**

1. T. M. Squires, S. R. Quake, Microfluidics: Fluid physics at the nanoliter scale. *Rev. Mod. Phys.* **77**(3), 977-1026 (2005).
2. F. Y. Ushikubo, F. S. Birribilli, D. R. B. Oliveira, R. L. Cunha, Y- and T-junction microfluidic devices effect of fluids. *Microfluid Nanofluid* **17**, 711–720 (2014).
3. T. Cubaud, T. G. Mason, Capillary threads and viscous droplets in square microchannels. *Physics of Fluids* **20**, 053302 (2008).
4. Y. Zhao, G. Chen, Q. Yuan, Liquid–liquid two-phase flow patterns in a rectangular microchannel. *AIChE Journal* **52**(12), 4052-4060 (2006).
5. A. Pohar, M. Lakner, I. Plazl, Parallel flow of immiscible liquids in a microreactor modeling and experimental study. *Microfluid Nanofluid* **12**, 307–316 (2012).
6. A. Gupta, S. M. S. Murshed, R. Kumar, Droplet formation and stability of flows in a microfluidic T-junction. *Appl. Phys. Lett.* **94**, 164107 (2009).
7. Y. Kim *et. al*., On-demand electrohydrodynamic jetting with meniscus control by a piezoelectric actuator for ultra-fine patterns. *J. Micromech. Microeng.* **19** 107001 (2009).
